# Mapping the Maxillary Artery and Lateral Pterygoid Muscle Relationship: Insights from Radiological and Meta-Analytic Evidence

**DOI:** 10.3390/medicina61071201

**Published:** 2025-06-30

**Authors:** Maria Piagkou, George Triantafyllou, Panagiotis Papadopoulos-Manolarakis, Fotis Demetriou, George Tsakotos, Łukasz Olewnik, Fabrice Duparc

**Affiliations:** 1 Department of Anatomy, Faculty of Health Sciences, School of Medicine, National and Kapodistrian University of Athens, 11527 Athens, Greece; georgerose406@gmail.com (G.T.); p.papado89@gmail.com (P.P.-M.); fotisdemetriou2000@gmail.com (F.D.); gtsakotos@gmail.com (G.T.); 2 “VARIANTIS” Research Laboratory, Department of Clinical Anatomy, Masovian Academy in Płock, 90 402 Płock, Poland; lukaszolewnik@gmail.com; 3 Department of Neurosurgery, General Hospital of Nikaia-Piraeus, 18454 Nikaia, Greece; 4 Department of Clinical Anatomy, Masovian Academy in Płock, 90 402 Płock, Poland; 5 Department of Anatomy, Faculty of Medicine-Pharmacy, University of Rouen-Normandy, 76183 Rouen, France; fabrice.duparc@univ-rouen.fr

**Keywords:** maxillary artery, lateral pterygoid muscle, variation, radiologic anatomy, infratemporal fossa

## Abstract

*Background/Objectives*: Variations in the course of the maxillary artery (MA) relative to the lateral pterygoid muscle (LPM) pose critical challenges in surgical, anesthetic, and interventional procedures involving the infratemporal fossa (ITF). These variations can increase the risk of hemorrhage, nerve injury, or incomplete anesthesia. The present study aimed to elucidate the topographic relationship between the MA and LPM by combining high-resolution radiological imaging with a comprehensive analysis of anatomical literature. *Materials and Methods*: A retrospective review of 250 brain computed tomography angiographies (CTAs), totaling 500 sides, was conducted to classify the MA course as lateral (superficial), medial (deep), or intramuscular. Additionally, a systematic review and meta-analysis of 32 eligible studies—including 5938 arteries—was performed following PRISMA 2020 and Evidence-Based Anatomy (EBA) guidelines. Study quality and risk of bias were assessed using the Anatomical Quality Assurance (AQUA) tool. *Results*: In the imaging cohort, the MA coursed lateral to the LPM in 64.2% of sides, medial in 29.6%, and through the muscle fibers in 6.2%. A rare temporalis-traversing variant was identified in 3.0% of cases. Bilateral symmetry was observed in 77.6% of patients. Meta-analytic findings indicated a pooled prevalence of 79.6% for the lateral course, 19.9% for the medial course, and 0.01% for the intramuscular course. Cadaveric studies and Asian populations showed a higher incidence of lateral variants, while imaging-based studies more frequently detected medial and transmuscular paths. *Conclusions*: While the MA most often follows a lateral course relative to the LPM, clinically significant variation—including medial, intramuscular, and temporalis-traversing routes—exists. These variants complicate access during maxillofacial surgery, TMJ procedures, and regional anesthesia. Findings emphasize the importance of individualized preoperative vascular mapping to improve procedural safety and outcomes in the ITF.

## 1. Introduction

The maxillary artery (MA), a major terminal branch of the external carotid artery, provides vascular supply to deep facial structures, including the maxilla, mandible, muscles of mastication, dura mater, and nasal cavity. As it traverses the infratemporal fossa (ITF), its anatomical course, particularly concerning the lateral pterygoid muscle (LPM), becomes clinically significant due to its proximity to vital neurovascular structures and common surgical corridors. This relationship is especially critical during interventions involving the mandibular nerve, temporomandibular joint (TMJ), or maxillofacial skeleton, where even minor variations in arterial trajectory can influence both procedural risk and outcome. The MA exhibits considerable topographical variation concerning the LPM [1,2]. This spatial variability has important clinical implications, especially in oral and maxillofacial surgery, interventional radiology, and regional anesthesia. Traditionally, the MA is described as either coursing laterally (superficial) or medially (deep) to the LPM, although a less common variant includes passage through the muscle itself [3]. These anatomical differences present potential challenges for procedures, such as mandibular nerve blocks, endovascular interventions, and botulinum toxin injections for oromandibular dystonias. Complications, including hemorrhage and unintended vascular injury, may arise if the arterial course is not identified preoperatively [3]. Despite routine procedures involving the infratemporal regions, such as mandibular nerve blocks, TMJ surgery, and endovascular interventions, comprehensive data on the prevalence and distribution of MA trajectories remain inconsistent across the literature. Variability in study design, population demographics, imaging resolution, and classification criteria has led to fragmented and sometimes contradictory findings, complicating the establishment of standardized anatomical references.

Ottone et al. [3] conducted a systematic review and meta-analysis utilizing the Anatomical Quality Assurance (AQUA) checklist to enhance anatomical precision within clinical settings. The analysis synthesizes findings from diverse cadaveric and radiological studies, thus providing a consolidated perspective on the relationship between MA and LPM. This introduction lays the groundwork for discussing the meta-analytic findings and their implications for surgical safety and anatomical education.

Despite decades of anatomical investigation, the prevalence of the MA-LPM co-variants remains inconsistent. Studies vary in methodology, population, and imaging resolution, leading to a fragmented understanding of the MA–LPM relationship. Cadaveric studies, while detailed, are limited by sample preservation and regional bias, whereas imaging studies provide contemporary, in vivo insights but are fewer in number. Given the growing reliance on radiologically guided procedures and the need for accurate vascular mapping, high-resolution imaging data and a comprehensive evidence synthesis are urgently needed. The significance of the MA–LPM relationship is heightened in procedures targeting the infratemporal region, such as temporomandibular joint surgeries and interventions involving the mandibular nerve, as well as in the interpretation of advanced imaging. There are also implications for pain syndromes and reconstructive procedures.

This study aims to (1) characterize the topographical variability of the MA to the LPM, utilizing computed tomography angiography (CTA) within a contemporary clinical cohort, and (2) to conduct a systematic review and meta-analysis of the extant literature to quantify the prevalence of each topographical variant across diverse populations and methodologies. Collectively, these data aspire to elucidate anatomical norms, underscore surgical risk zones, and facilitate enhanced planning in invasive procedures concerning the craniofacial region.

## 2. Materials and Methods

### 2.1. Computed Tomography Angiography (CTA) Study

Two hundred and fifty brain CTAs were retrospectively and randomly selected to investigate the topographic relationship between the MA and the LPM. The study population consisted of 138 male and 112 female patients, with a mean age of 59.5 ± 14.5 years. Imaging was performed at the General Hospital of Nikaia–Piraeus (*Athens*, *Greece*) using a 128-slice helical scanner (SOMATOM go.Top, Siemens Healthineers, Erlangen, Germany), with participants in the supine position and neutral head alignment. Each scan followed an intravenous administration of 60 mL of iodinated contrast medium (30% concentration) delivered at a 4.0–4.5 mL/s flow rate.

**Figure 1:**
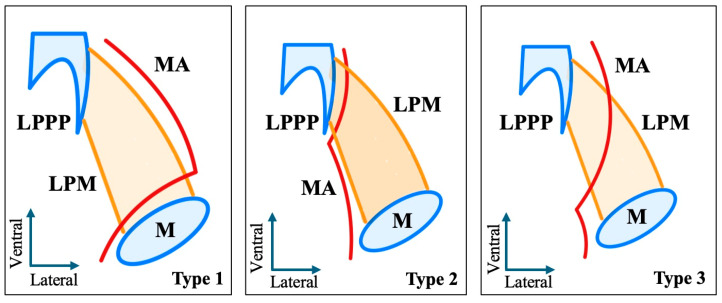
Schematic representation of the courses of the maxillary artery (MA) in relation to the lateral pterygoid muscle (LPM) in axial view. Type 1—superficial/lateral course, Type 2—deep/medial course, Type 3—muscular course. LPPP—lateral pterygoid process plate, M- maxilla.

The study protocol was approved by the hospital’s institutional review board (*Protocol No. 56485*; *approval date: 13 November 2024*). Three reviewers (MP, GTr, FDe) analyzed anonymized images independently using Horos software (*Horos Project*, *New York*, *NY*, *USA*). Assessments included multiplanar reconstructions (axial, coronal, sagittal) and three-dimensional volume renderings. The anatomical relationship between the MA and LPM was classified based on criteria adapted from previous morphological studies [4] (Figure 1). Any discrepancies were resolved by consensus in consultation with senior authors.

Despite the high resolution of the CTAs and rigorous assessment protocols, certain limitations inherent to radiologic imaging must be acknowledged. Vascular overlap, motion artifacts, and patient-specific morphological and/or topographical variations occasionally made it challenging to distinguish between medial and intramuscular courses, particularly in complex or crowded infratemporal configurations. Additionally, the absence of intraoperative or cadaveric correlation may have limited the confirmation of subtle variants. Three experienced reviewers independently assessed all images to mitigate interpretation bias, with inter-observer agreement initially calculated at 91.2%. Remaining disagreements were adjudicated through consensus discussions, ensuring consistency in classification. This multi-reviewer approach aimed to improve reliability and reduce the subjectivity often associated with anatomical interpretations in radiological datasets.

### 2.2. Systematic Review with Meta-Analysis

This component adhered to the Evidence-Based Anatomy (EBA) Workgroup recommendations for conducting anatomical meta-analyses [5] and followed the PRISMA 2020 guidelines for the transparent and systematic reporting of reviews [6]. These frameworks ensured methodological rigor, including comprehensive literature search strategies, structured data extraction, and standardized bias assessment. Although the protocol was not preregistered in PROSPERO or other systematic review registries, all procedures were predefined and consistently applied throughout the review process. The decision not to register was based on the study’s specific anatomical focus, which falls outside the scope of many clinical trial registries. Nevertheless, all efforts were made to maintain reproducibility and transparency, aligning with best practices in anatomical synthesis.

A literature search was conducted through PubMed, Google Scholar, Scopus, and Web of Science, from January 2025 to May 2025. Search terms included combinations of “maxillary artery”, “lateral pterygoid muscle”, “relationship”, “variation”, “cadaveric study”, and “imaging study.” Manual searches were also performed in leading anatomical journals: *Annals of Anatomy*, *Clinical Anatomy*, *Journal of Anatomy*, *Anatomical Record*, *Surgical and Radiologic Anatomy*, *Folia Morphologica*, *European Journal of Anatomy*, *Anatomical Science International*, *Anatomy and Cell Biology*, and *Morphologie*. Reference lists were screened for additional eligible studies, and the relevant gray literature was reviewed. Inclusion criteria required studies to report quantitative data on the variants of the MA to the LPM. Case reports, textbooks, books’ chapters, animal studies, conference abstracts, and studies without extractable or relevant prevalence data were excluded.

Two independent reviewers (GTr, FDe) screened all titles, abstracts, and full texts and extracted data into standardized Microsoft Excel templates. Discrepancies were resolved by consensus with senior reviewers. Methodological quality and risk of bias were evaluated using the Anatomical Quality Assurance (AQUA) tool [7].

All statistical analyses were conducted using R software (version 4.3.2, The R Foundation, Kaysville, United States of America), incorporating the “meta” and “metafor” packages for meta-analytic modeling and visualization. A single investigator (GTr) performed statistical procedures to ensure consistency in data handling and interpretation.

Pooled prevalence rates of MA morphological–topographical variants were calculated using a random-effects model with inverse variance weighting to account for expected heterogeneity across studies. The Freeman–Tukey double arcsine transformation stabilized variances, particularly for proportions near 0 or 1. Between-study heterogeneity (τ^2^) was estimated using the DerSimonian–Laird method, while 95% confidence intervals were derived using the Jackson method.

Untransformed raw means were analyzed for continuous variables, such as mean vessel diameters. Between-study variance was calculated using the restricted maximum-likelihood method, and the Q-profile approach was applied to construct confidence intervals for τ^2^ and τ, enhancing the precision of heterogeneity estimates.

The subgroup analyses explored differences based on study type (cadaveric vs. imaging-based) and geographic origin. Heterogeneity across studies was evaluated using Cochran’s Q test (with a significance threshold of *p* < 0.10) and quantified using Higgins’ I^2^ statistic, categorized as follows: 0–40% (low), 30–60% (moderate), 50–90% (substantial), and 75–100% (considerable).

A DOI plot and the LFK index were used to assess potential publication bias and small-study effects, as recommended for anatomical meta-analyses involving prevalence data [8]. This multifaceted analytical approach ensured methodological rigor and transparency throughout the synthesis process.

## 3. Results

### 3.1. Original Radioanatomical Study

**Figure 2:**
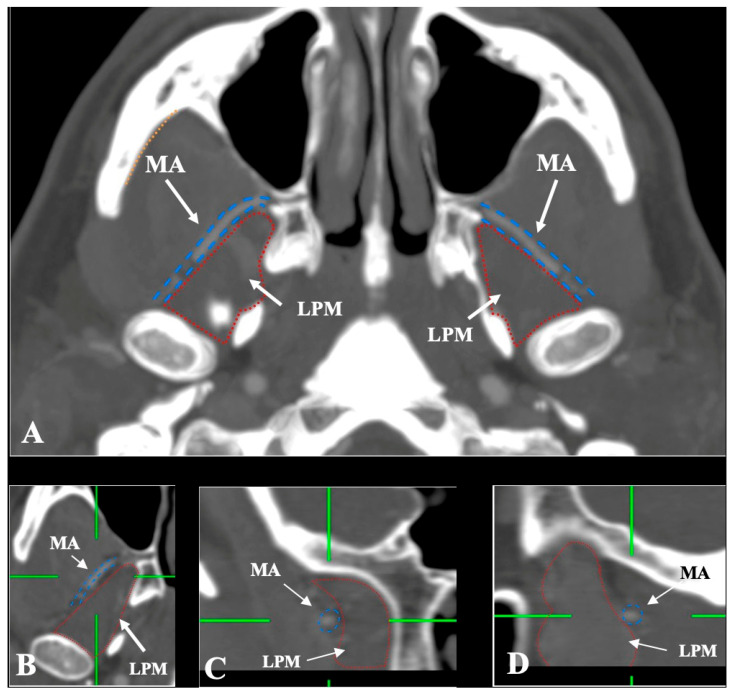
(**A**–**D**) The superficial/lateral course of the maxillary artery (MA) (blue border) in relation to the lateral pterygoid muscle (LPM) (red border) in axial, coronal, and sagittal slices.

**Figure 3:**
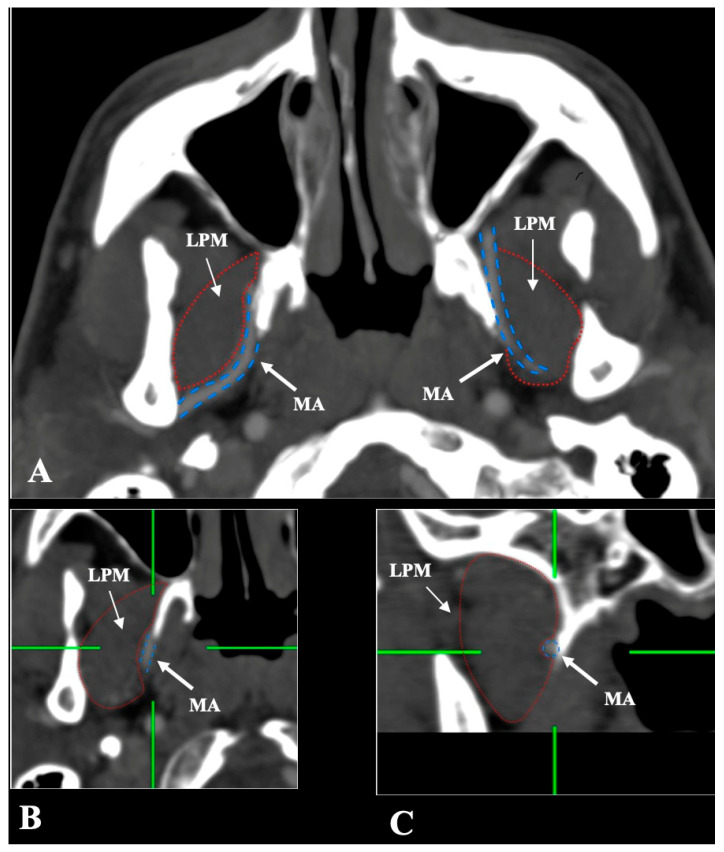
(**A**–**C**) The deep/medial course of the maxillary artery (MA) (blue border) in relation to the lateral pterygoid muscle (LPM) (red border) in axial, coronal, and sagittal slices.

**Figure 4:**
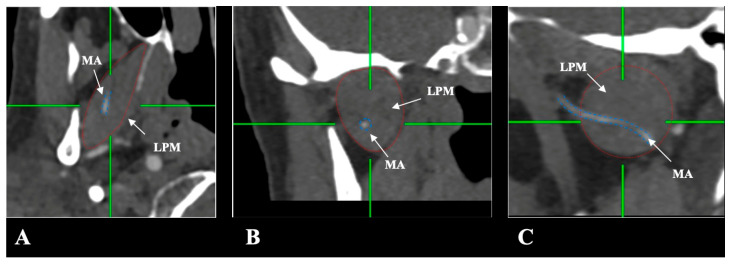
(**A**–**C**) The intramuscular course of the maxillary artery (MA) (blue border) in relation to the lateral pterygoid muscle (LPM) (red border) in axial, coronal, and sagittal slices.

The MA and LPM were identified bilaterally in all 250 patients, yielding 500 sides (100% dataset completeness). Three distinct anatomical relationships between the MA and the LPM were observed:

- Lateral (superficial) to the LPM: Detected in 321 sides (64.2%). This configuration was consistently visualized in axial, coronal, and sagittal planes (Figure 2).
- Medial (deep) to the LPM: Identified in 148 sides (29.6%), best appreciated on axial and coronal reconstructions (Figure 3).
- Intramuscular (transversing the LPM fibers): Found in 31 sides (6.2%), clearly depicted in all planes (Figure 4).

**Figure 5:**
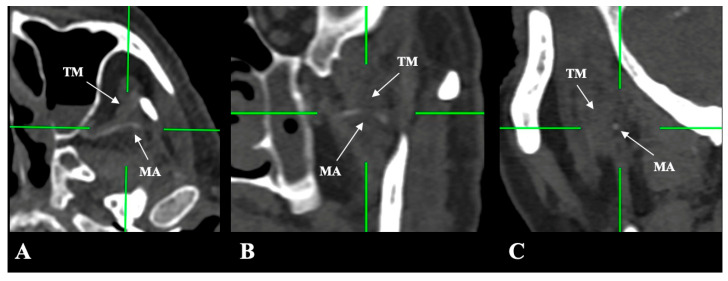
Superficial and lateral course of the maxillary artery (MA), deviating through the temporalis muscle (TM), as shown in the axial slice (**A**), coronal (**B**), and sagittal (**C**).

An infrequent variant was also observed: in 15 sides (3.0%), the MA initiated with one of those mentioned above coursed and then deviated from its usual path to traverse the temporalis muscle (Figure 5).

No significant differences in MA position were found based on patient sex or side (Table 1). The lateral course was slightly more frequent on the left side (65.6%) compared to the right (62.8%), but this difference was not significant (*p* = 0.320). Similarly, by sex, the lateral trajectory occurred in 63.8% of males and 64.7% of females (*p* = 0.117). The medial variant appeared in 29.6% of sides with near-identical left–right symmetry (74 per side) and showed negligible sex differences (28.6% in males vs. 30.8% in females). The intramuscular variant showed a slightly higher frequency in males (7.6% vs. 4.5%) and on the right side (7.6% vs. 4.8%), but these differences also lacked statistical significance.

Bilateral symmetry in MA–LPM relationships was noted in 194 patients (77.6%), with asymmetrical patterns observed in 56 patients (22.4%) (Table 2). The most common symmetrical configuration was bilateral lateral course (*n* = 136; 54.4%), followed by bilateral medial (*n* = 50; 20.0%) and bilateral intramuscular (*n* = 8; 3.2%). Among asymmetrical patterns, the most frequent combination was lateral on one side and medial on the other (*n* = 27; 10.8%). Fewer cases involved a mix of intramuscular and either lateral or medial positioning. These findings emphasize the predominance of symmetry in MA anatomy but also underscore the need for individualized assessment due to non-negligible asymmetry rates.

### 3.2. Systematic Review with Meta-Analysis

**Figure 6:**
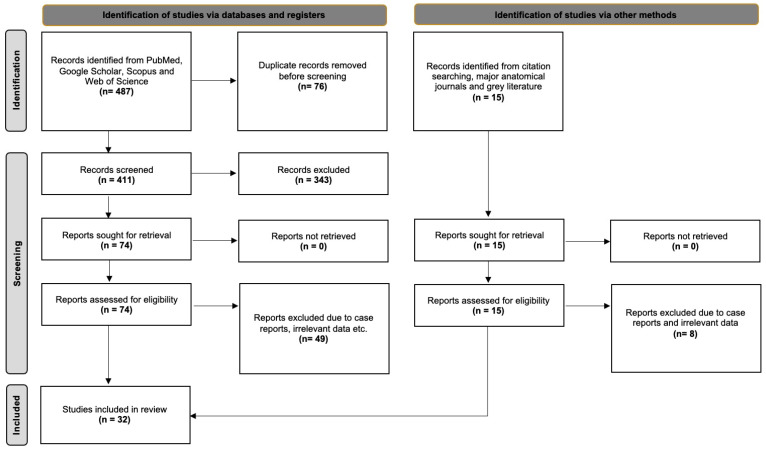
Search the current literature analysis according to PRISMA 2020 guidelines [6].

The initial search retrieved 487 articles. After screening and deduplication, 32 studies met the inclusion criteria and were incorporated into the meta-analysis (Figure 6).

These studies included 5938 arteries: 27 cadaveric and 5 imaging-based. Studies originated from Asia (*n* = 16), Europe (*n* = 10), the Americas (*n* = 5), and Africa (*n* = 1). Risk of bias, assessed using the AQUA checklist, was low in 18 studies and high in 14. Imaging studies generally demonstrated lower bias due to standardized acquisition protocols, while older cadaveric studies showed greater methodological variability (Table 3).

**Table 3:** Characteristics of studies included in the meta-analysis of maxillary artery–lateral pterygoid muscle relationship.

Study	Year	Population	Type of Study	No. of Arteries	Risk of Bias
Thomson [9]	1890	Europe	Cadaveric	447	Low
Adachi [10]	1928	Asia	Cadaveric	331	High
Fujita [11]	1932	Asia	Cadaveric	119	High
Kijima [12]	1932	Asia	Cadaveric	20	High
Lurje [13]	1946	Europe	Cadaveric	200	High
Lasker et al. [14]	1951	America	Cadaveric	216	High
Takarada [15]	1958	Asia	Cadaveric	120	High
Krizan [16]	1960	Europe	Cadaveric	200	Low
Ikakura [17]	1961	Asia	Cadaveric	160	High
Skopakoff [18]	1968	Europe	Cadaveric	180	High
Czerwinski [19]	1981	Europe	Cadaveric	240	Low
Iwamoto et al. [20]	1981	Asia	Cadaveric	158	Low
Sashi [21]	1989	Asia	Cadaveric	100	Low
Suwa et al. [22]	1990	Asia	Cadaveric	278	Low
Pretteklieber et al. [23]	1991	Europe	Cadaveric	204	Low
Tsuda [24]	1991	Asia	Cadaveric	339	Low
Fujimura et al. [25]	2006	Asia	Cadaveric	12	High
Isolan et al. [26]	2007	America	Cadaveric	16	High
Orbay et al. [27]	2007	Europe	Cadaveric	16	High
Hussain et al. [28]	2008	America	Cadaveric	88	Low
Dennison et al. [29]	2009	America	Cadaveric	104	Low
Balcioglu et al. [30]	2010	Asia	Cadaveric	34	Low
Otake et al. [31]	2011	Asia	Cadaveric	28	Low
Gulses et al. [32]	2012	Asia	Imaging	418	Low
Maeda et al. [33]	2012	Asia	Cadaveric	208	Low
Joo et al. [34]	2013	Asia	Cadaveric	20	High
Hwang et al. [35]	2014	Asia	Imaging	200	Low
Alvernia et al. [36]	2017	America	Cadaveric	12	High
Makosa et al. [37]	2022	Africa	Cadaveric	30	High
Schonegg et al. [38]	2022	Europe	Imaging	600	Low
Albu et al. [4]	2025	Europe	Imaging	340	Low
Current Study	2025	Europe	Imaging	500	-

Meta-analysis estimates for the anatomical relationship between the MA and LPM are as follows:

- Lateral (superficial) course: 79.61% (95% CI: 73.53–85.11);
- Medial (deep) course: 19.94% (95% CI: 14.57–25.88);
- Intramuscular course: 0.01% (95% CI: 0.00–0.30).

**Figure 7:**
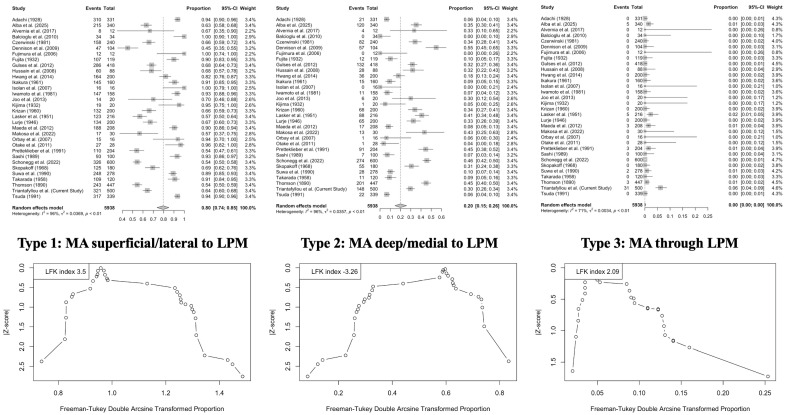
Results from statistical meta-analysis are presented with Forest plots (**top**) and DOI plots with LFK indices (**bottom**), illustrating the pooled prevalence and publication bias analysis of the three anatomical courses of the maxillary artery (MA) in relation to the lateral pterygoid muscle (LPM) [4,9,10,11,12,13,14,15,16,17,18,19,20,21,22,23,24,25,26,27,28,29,30,31,32,33,34,35,36,37,38].

Figure 7 graphically illustrates these data, suggesting that the lateral configuration is overwhelmingly dominant across anatomical studies.

*Geographic origin* significantly influenced the prevalence of MA variants. The lateral course was most frequent in studies from Asia (90.79%), compared to Europe (62.93%), the Americas (67.70%), and Africa (56.67%) (*p* < 0.0001). The medial course was inversely more prevalent outside of Asia. No significant regional differences were detected for the intramuscular variant (*p* = 0.2335) (Table 4).

Cadaveric studies showed a significantly higher prevalence of the lateral MA course (82.11%) than imaging studies (66.54%) (*p* = 0.0046). In contrast, imaging studies reported a higher frequency of the medial course (31.89%) compared to cadaveric studies (17.63%) (*p* = 0.0107). The prevalence of the intramuscular variant did not differ significantly between modalities (*p* = 0.6002). These findings suggest that population-based anatomical variability and methodological factors influence the apparent distribution of MA–LPM relationships, reinforcing the need for contextual interpretation of the prevalence data in clinical applications.

## 4. Discussion

The ITF presents a wide morphology of muscular [39,40], arterial [3], osseous [41,42,43], and neural variants [44,45]. Our findings reinforce the ITF’s complex and variable vascular anatomy, highlighting the importance of personalized anatomical assessment in clinical practice.

This integrated radiological and meta-analytic study offers a comprehensive and updated assessment of the anatomical relationship between the MA and the LPM, a region crucial to maxillofacial surgery, regional anesthesia, and anatomical training. In temporomandibular joint (TMJ) surgery, the MA frequently courses near—or sometimes through—the TMJ capsule, particularly when following a deep path medial to the LPM [3,36]. This morphological (topographical) variant presents a significant bleeding risk during arthroplasty, discectomy, and open reduction [25,26]. The MA’s trajectory may influence the choice of surgical access to avoid vascular injury and support better postoperative outcomes [27,31].

In mandibular nerve blocks and related interventions, the MA lies in close proximity to the inferior alveolar and lingual nerves, both branches of the mandibular division (V3) [1,44]. Aberrant arterial paths can result in intravascular injections, increasing the risk of systemic toxicity and ineffective anesthesia [3,28]. Therefore, image-guided techniques—ultrasound (US) or CT—must account for the MA’s position relative to the LPM [32,35].

CTA and MRI are routinely employed to evaluate infratemporal lesions, vascular malformations, and tumor spread. Understanding the MA’s anatomical course is critical in differentiating between vascular tumors (e.g., *juvenile nasopharyngeal angiofibroma*), arteriovenous malformations, and iatrogenic pseudoaneurysms [3,36,46]. This knowledge is especially vital in advanced navigation-assisted surgeries, including robotic TMJ interventions, where precise mapping of the MA–LPM relationship ensures safer intraoperative planning [25,31].

A deep MA trajectory may also lead to the neurovascular compression of V3 branches, contributing to trigeminal neuralgia or myofascial pain dysfunction syndrome [44,45,47]. Vascular contact with nerve fibers has been proposed as a trigger for neuropathic facial pain [3,44]. During oncologic resections or trauma repair, the MA often serves as a recipient vessel for microvascular free flap reconstruction. Accurate anatomical knowledge facilitates reliable anastomosis, reduces hemorrhagic complications, and guides pedicle selection [26,36].

Finally, the MA’s course may be altered in craniofacial developmental disorders or congenital vascular anomalies. These variations are highly relevant in pediatric TMJ ankylosis surgeries and craniofacial distraction osteogenesis, where vascular mapping is essential for procedural success and safety [48,49].

In our imaging cohort, the MA coursed laterally to the LPM in 64.2% of cases, medially in 29.6%, and through the muscle in 6.2%. Additionally, a rare variant involving the temporalis muscle was identified in 3% of sides. These proportions are broadly consistent with prior radiographic studies but fall below the pooled prevalence of 79.61% for the lateral variant reported in our meta-analysis. The discrepancy highlights the influence of population characteristics and methodological approaches on reported prevalence.

Consistent with the previous literature, the lateral (superficial) course was the most frequent configuration. Subgroup analyses in our meta-analysis confirmed that cadaveric studies (Table 4) and studies from Asian populations (Table 4) reported significantly higher frequencies of lateral MA courses. Conversely, imaging-based investigations, including ours, identified higher proportions of medial and intramuscular trajectories, likely reflecting the improved visualization of soft tissue interfaces and vascular paths (Table 4).

Recent research highlights the MA’s variability in the LPM and clinical implications. Ottone et al. [3] used the AQUA checklist in a systematic review, showing global variability and the need for population-specific anatomical mapping. Collectively, these studies advocate for individualized, radiologically guided planning in craniofacial procedures to enhance safety and effectiveness. Understanding the MA’s trajectory is essential for safe navigation during sagittal split osteotomies, coronoidectomies, TMJ surgeries, and inferior alveolar nerve decompressions. While the lateral course presents the most predictable configuration, the intramuscular and trans-temporalis variants introduce additional surgical risk, particularly for hemorrhagic complications if not anticipated during planning. Notably, this study’s intramuscular and temporalis-traversing variants correspond with findings from high-resolution imaging studies such as those by Dumitru et al. [46] and Verma et al. [47], which reported trans-temporalis MA pathways in up to 5.6% of sides. These findings suggest that while infrequent, these variants may be underrepresented in dissection-based studies due to methodological constraints or tissue distortion.

The topographic variability of the MA likely reflects the embryological persistence of vascular loops and the influence of surrounding mesenchymal structures derived from the first pharyngeal arch [48]. Variations in the LPM—ranging from single- to multi-headed configurations—further modulate the available anatomical corridors for vascular passage [40,49]. The intramuscular course, in particular, may result from both vascular deviation and muscular heterotopia. Structural heterogeneity in the origin and insertion of the LPM can create corridors or resistance planes, thereby shaping arterial paths during development.

The spatial intimacy between the MA and branches of the mandibular nerve (V3) suggests the possibility of dynamic neurovascular compression syndromes. Piagkou et al. [44] documented that variations in the MA course may contribute to facial pain, mandibular nerve entrapment, or temporomandibular dysfunction, particularly when the artery pierces or abuts muscular and neural structures. Moreover, the MA supplies key territories innervated by V3, making its trajectory relevant in diagnostic nerve blocks and interventional procedures. Aberrant arterial positioning can complicate or contraindicate specific anesthetic approaches and influence the safety of deep facial injections.

A few limitations should be acknowledged. First, our retrospective imaging study was conducted at a single center, potentially limiting generalizability. Second, the meta-analysis relied on studies with heterogeneous methods, variable resolution, and differing dissection protocols. Some of the included studies, particularly older cadaveric reports, carried a higher risk of bias, despite being accounted for in subgroup analyses. Additionally, pediatric anatomy and MA branches were not assessed, leaving areas for future exploration.

Future investigations should prioritize prospective, multicenter studies incorporating advanced imaging techniques such as three-dimensional computed tomography angiography (3DCTA), magnetic resonance angiography (MRA), and augmented or mixed reality visualization tools. These modalities provide superior spatial resolution and interactivity, facilitating more precise mapping of the MA in relation to the LPM. Concurrently, developmental studies examining the embryological basis and morphogenetic pathways of MA–LPM variants—especially when associated with muscle architecture or craniofacial growth patterns—could offer new insights into their anatomical and clinical significance. Furthermore, integrating anatomical data with clinical outcomes, including pain syndromes, anesthetic efficacy, and intraoperative complications, would greatly enhance the translational value of such research. This patient-centered, anatomy-informed approach can potentially improve preoperative planning, reduce procedural risks, and tailor interventions in craniofacial and neurosurgical practice.

## 5. Conclusions

The MA most frequently courses laterally to the LPM, but notable variation exists, including medial, intramuscular, and trans-temporalis pathways. Recognition of these variants is essential for safe and effective surgical, anesthetic, and interventional procedures involving the infratemporal region. Incorporating high-resolution imaging, as well as population and methodological variability awareness, can help clinicians anticipate and manage these anatomical complexities. This study assessed the possible courses through CTA with a slice thickness of 0.8 mm and depicted that this was an easy and safe option to identify morphological variability. Routine preoperative imaging should be considered standard in complex maxillofacial interventions to detect atypical MA paths.

## Figures and Tables

**Table 1:** The relationship between the maxillary artery (MA) and the lateral pterygoid muscle (LPM) is analyzed by side and sex.

MA-LPM Relationship	Total N = 500 (%)	Left *n* = 250 (%)	Right *n* = 250 (%)	*p*-Value	Males *n* = 276 (%)	Females *n* = 224 (%)	*p*-Value
Lateral/Superficial	321 (64.2)	164 (65.6)	157 (62.8)	0.320	176 (63.8)	145 (64.7)	0.117
Medial/Deep	148 (29.6)	74 (29.6)	74 (29.6)		79 (28.6)	69 (30.8)	
Through	31 (6.2)	12 (4.8)	19 (7.6)		21 (7.6)	10 (4.5)	

**Table 2:** The relationship between the maxillary artery (MA) and the lateral pterygoid muscle (LPM) is defined according to laterality.

MA-LPM Relationship	Lateral/Superficial *n* (%)	Medial/Deep *n* (%)	Through *n* (%)
Lateral/Superficial	136 (54.4)	19 (7.6)	2 (0.8)
Medial/Deep	22 (8.8)	50 (20)	2 (0.8)
Through	6 (2.4)	5 (2)	8 (3.2)

**Table 4:** Subgroup analysis of maxillary artery (MA) trajectory relative to the lateral pterygoid muscle (LPM) by geographic region and study type. Asterisks (*) indicate the statistically significant results.

Parameters	Superficial/Lateral	Deep/Medial	Through
Asia (*n* = 16)	90.79%	9.04%	0.00%
Europe (*n* = 10)	62.93%	35.66%	0.36%
Africa (*n* = 1)	56.67%	43.33%	0.06%
America (*n* = 5)	67.70%	31.80%	0.00%
*p*-value	<0.0001 *	<0.0001 *	0.2335
Cadaveric (*n* = 27)	82.11%	17.63%	0.00%
Imaging (*n* = 5)	66.54%	31.89%	0.73%
*p*-value	0.0046 *	0.0107 *	0.6002

## Data Availability

All the data are available upon reasonable request to the corresponding author (Professor Maria Piagkou—mapian@med.uoa.gr).
